# The number of the intraepithelial T cells correlate with the proliferation index in human bulbourethral gland epithelium

**DOI:** 10.1016/j.heliyon.2022.e11658

**Published:** 2022-11-17

**Authors:** Tatiana Boronikhina, Gennadii Piavchenko, Artem Venediktov, Ekaterina Kustavinova, Svetlana Mukhamedova, Natalia Kartashkina, Sergey Kuznetsov, Igor Meglinski, Alexander Yatskovskiy

**Affiliations:** a Department of Histology, Cytology, and Embryology, I.M. Sechenov First Moscow State Medical University (Sechenov University), Moscow, Russia; b Opto-Electronics and Measurement Techniques, Faculty of Information and Electrical Engineering, University of Oulu, Oulu, Finland; c College of Engineering and Physical Sciences, Aston University, Birmingham, UK

**Keywords:** Epithelial proliferation, Human bulbourethral glands, Lymphocytic infiltration, Proliferating cell nuclear antigen, T cells

## Abstract

**Background:**

Our study has immunohistochemically examined T cells localization and number as well as proliferative activity level for the bulbourethral gland epithelium in men of different ages, using monoclonal antibodies against CD45RO and proliferating cell nuclear antigen (PCNA).

**Results:**

We have found that the T cells have been localized mainly in excretory ducts epithelium of the glands in any age group, meanwhile their relative number varies with age. The excretory ducts epithelium has shown a high proliferative activity when in acini the PCNA index has been low. Postnatal dynamics of the epithelium proliferative activity positively correlates with age-related density fluctuations in lymphocytic infiltration of the glands.

**Conclusions:**

We consider that intraepithelial T cells may contribute to the regulation of epithelial cells proliferation in the bulbourethral glands.

## 1 Introduction

Among the organs of the male reproductive system, the bulbourethral glands remain the least studied [1], especially compared to the prostate, numerous studies of which are dictated by the need to find better approaches to the treatment of such common nosologies as benign prostatic hyperplasia (BPH) and prostate cancer. The lack of proper attention to the study of bulbourethral glands may be due to the rarity of the development of pathological processes in them, including neoplastic ones [1, 2]. The most common lesion of the bulbourethral glands is a syringocele or cyst of the main duct, the pathogenesis of which is due to changes in the proliferative activity of the glandular epithelium and disruption of cellular homeostasis [3, 4]. This circumstance makes it relevant to study the possible mechanisms of regulatory influences on proliferative processes in the epithelium of the bulbourethral glands. Systemic regulators of the mitotic activity of the epithelium of the accessory glands of the male genital tract are androgens, whose action is mediated through the suppression of apoptosis [5, 6]. Local regulators, among others, can be intraepithelial T lymphocytes, whose participation in epithelial proliferation has been established for a number of organs [7, 8, 9, 10, 11], including the prostate [12]. Because androgen blood levels in the male body are subject to age-related fluctuations, this should inevitably affect the intensity of proliferation of the epithelial cells. In this regard, the ratio of systemic and local proliferation regulators in different age periods raises an undoubted interest.

T cells predominate among leukocytes infiltrating human bulbourethral gland epithelium [13], where they are supposed to provide antitumor protection, as well as to form a local immunological barrier against antigens of sperm and urogenital mucous membranes microbiota [14, 15].

Based on assumption of the possible involvement of T cells in the regulation of proliferation, this study has been designed to identify if there is a correlation between the number of intraepithelial T cells and proliferative activity of the bulbourethral gland epithelium in men of different age, considering that the obtained data is related to androgen level fluctuations.

## 2 Materials and methods

Bulbourethral glands have been taken from men of different age (beginning with infants and up to the age of ninety) who deceased not because of pelvic organs pathology or even due to unexpected non-disease related causes (40 cases in total). An informed consent was obtained for all the cases that were analyzed in the current research from the close relatives of the deceased person. The study has been approved by the Local Ethical Committee of Sechenov University on March 2019 (protocol #03–19). The cause of death was determined by forensic medical examination. The material has been classified in accordance with the following age periodization: infancy (10 days–1 year, n = 4), toddlers and early childhood (1–7 years, n = 3), late childhood (8–12 years, n = 3), early adolescence (13–16 years, n = 4), late adolescence (17–21 years, n = 4), early adulthood (22–35 years, n = 5), middle adulthood (36–60 years, n = 9), late adulthood/young old age (61–74 years, n = 5), and middle & oldest-old age (75–90 years, n = 3). Considering that a decrease in the level of testosterone and reproductive function changes are more prominent in men of middle adulthood, namely after 45 years [16], we have separately studied the cases from 36 to 45 years' group (n = 5) and from 46 to 60 years’ group (n = 4). Thus, 10 age groups have been examined in total. The glands have been fixed in 10% neutral formalin and embedded in paraffin. Immunohistochemical reactions have been carried out using monoclonal antibodies against CD45RO T cell antigen (DakoCytomation, Germany) and proliferating cell nucleus marker, PCNA (DakoCytomation, Germany).

CD45RO is an isoform of CD45 that is a transmembrane glycoprotein expressed on most nucleated cells of hemopoietic origin. CD45RO is quite specific for T cells, including most thymocytes, a subpopulation of resting T cells within both CD4 and CD8 subsets, mature, activated T cells and may also be present at lower levels on various myeloid cells.

PCNA (proliferating cell nucleus antigen) is a protein involved in DNA replication in the S period of the mitotic cycle as a co-factor of DNA polymerase. Expression level of PCNA is directly correlated with cell proliferative activity.

Deparaffinized sections were pretreated in a microwave in a pH 6.0 citrate buffer for 10 min at 750 W. The sections then have been incubated with the antibodies for 30 min at room temperature. The labeled streptavidin-biotin methods (LSAB; DakoCytomation, Germany) detection system, based on the biotin-streptavidin complex and peroxidase, has been used to reveal the reaction (sequential 10-minute incubations with biotinylated link antibody and peroxidase-labelled streptavidin). We have also used diaminobenzidine as a visualization substrate. After the reaction, the sections have been additionally stained with hematoxylin and embedded in a mounting medium. At the same time, all the necessary controls were carried out.

Quantitative analysis of T cells and epithelial proliferation index in the sections of the glands (7 μm thickness) has been performed with a light microscope under a 16 × 40 magnification. We analyzed 3 slides per each case, 6 fields of vision for every slide. The results were counted for each field of view, after that it was averaged in each case followed by every group averaging. In every slide, we have counted positively reactive cells per 1000 epitheliocytes in the bulbourethral gland acini, simple epithelium of intralobular ducts, and in stratified epithelium of interlobular ducts. Then we have determined the percentage of T cells and PCNA index among the counted cells. The normality of distribution assessment was carried out. After that we calculated the mean and standard error (M±SE) parameters for each case. Pearson's correlation coefficients (rxy) were computed. Statistical data processing has been carried out using Statistical Package for Social Studies software (SPSS version 24; IBM Corporation, Armonk, NY, USA) to compare variables. Student's test has been used to compare means. The level of significance was set at p < 0.05).

## 3 Results

**Figure 1:**
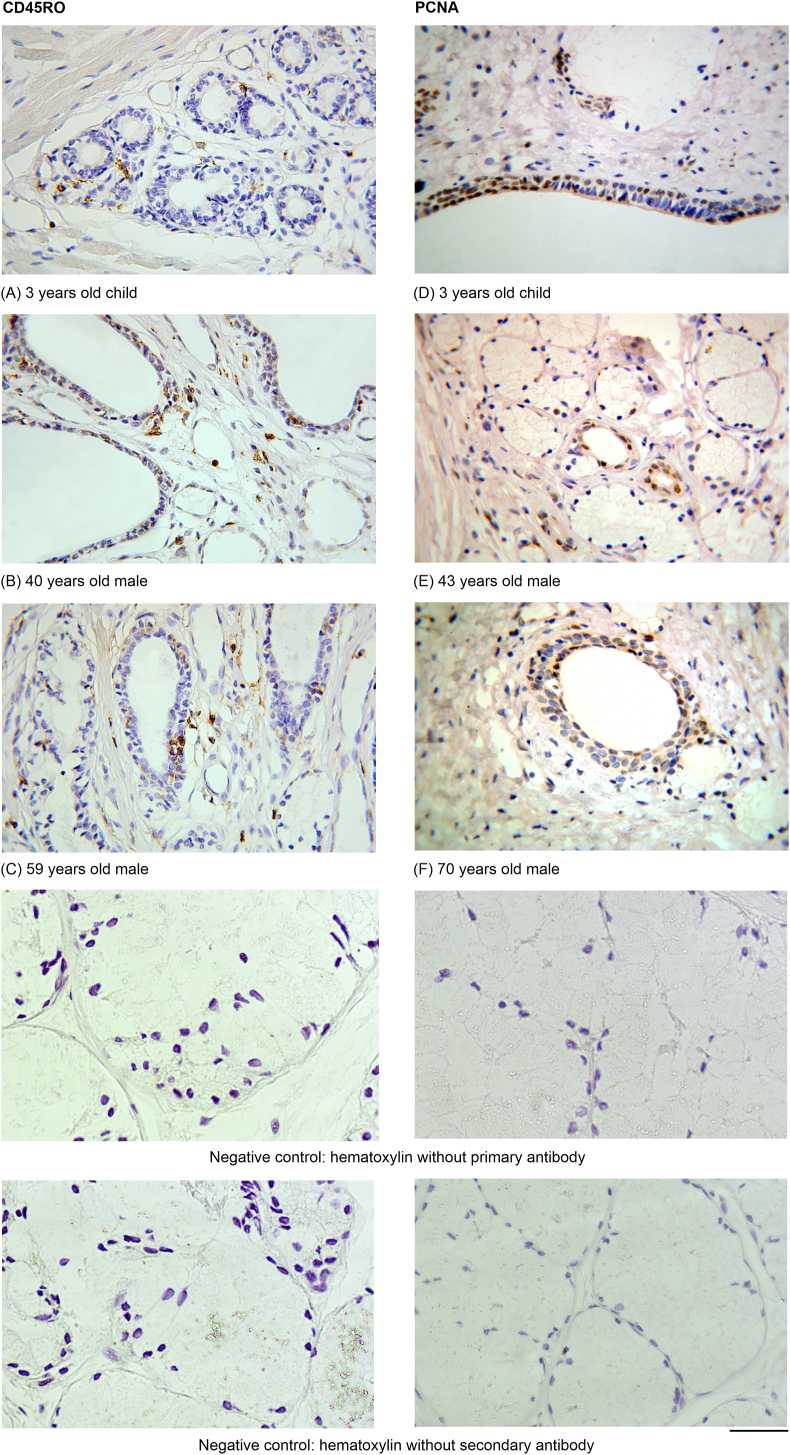
CD45RO-positive and PCNA-positive cells *(brown)* in bulbourethral glands' ducts and acini for different ages. Oc.20x, ob.63x. (A) 3 years old child: in the lobule with developing acini, there are positively colored cells among glandulocytes and in the surrounding connective tissue; (B) 40 years old male: there are some positively colored cells in the stratified epithelium of three interlobular ducts (*in the upper part*); in acini (*lower and on the right of ducts*), no positively colored cells are visualized; some positively colored cells are distributed in the surrounding connective tissue; (C) 59 years old male: there are some positively colored cells in the stratified epithelium of two interlobular ducts (*in the center and above it*); single positively colored cells are present in acini (*lower and on the right of ducts*); the surrounding connective tissue contains numerous positively colored cells. (D) 3 years old child: there are a lot of positively colored nuclei in the stratified epithelium of an interlobular duct (*in the bottom*); acini (*upper the duct*) display single positively colored nuclei; (E) 43 years old male: there are positively colored nuclei in the simple epithelium of two intralobular ducts (*in the center*); acini (*around the ducts*) are seen without colored nuclei; (F) 70 years old male: there are positively colored nuclei in the stratified epithelium of the interlobular duct (*in the center*); some positively colored nuclei are in the acinus linked with the duct. Negative controls for the staining of both markers including no primary antibody and no secondary antibody are presented.

Intraepithelial CD45RO-positive cells, which are found to be more numerous in the duct epithelium and less numerous in the acini, have been revealed in the bulbourethral glands of all studied age groups (Figure 1). The difference between the number of T cells in stratified epithelium of the interlobular ducts and in simple epithelium of the intralobular ducts have not been significant.

For the bulbourethral glands of all the studied age groups, PCNA index in stratified epithelium of interlobular ducts is higher than in simple epithelium of intralobular ducts. Proliferative activity of cells in the secretory portions is less than in the ducts. Glandular cells with PCNA-positive nuclei have been found mainly in acini adjacent to the ducts (Figure 1).

**Figure 2:**
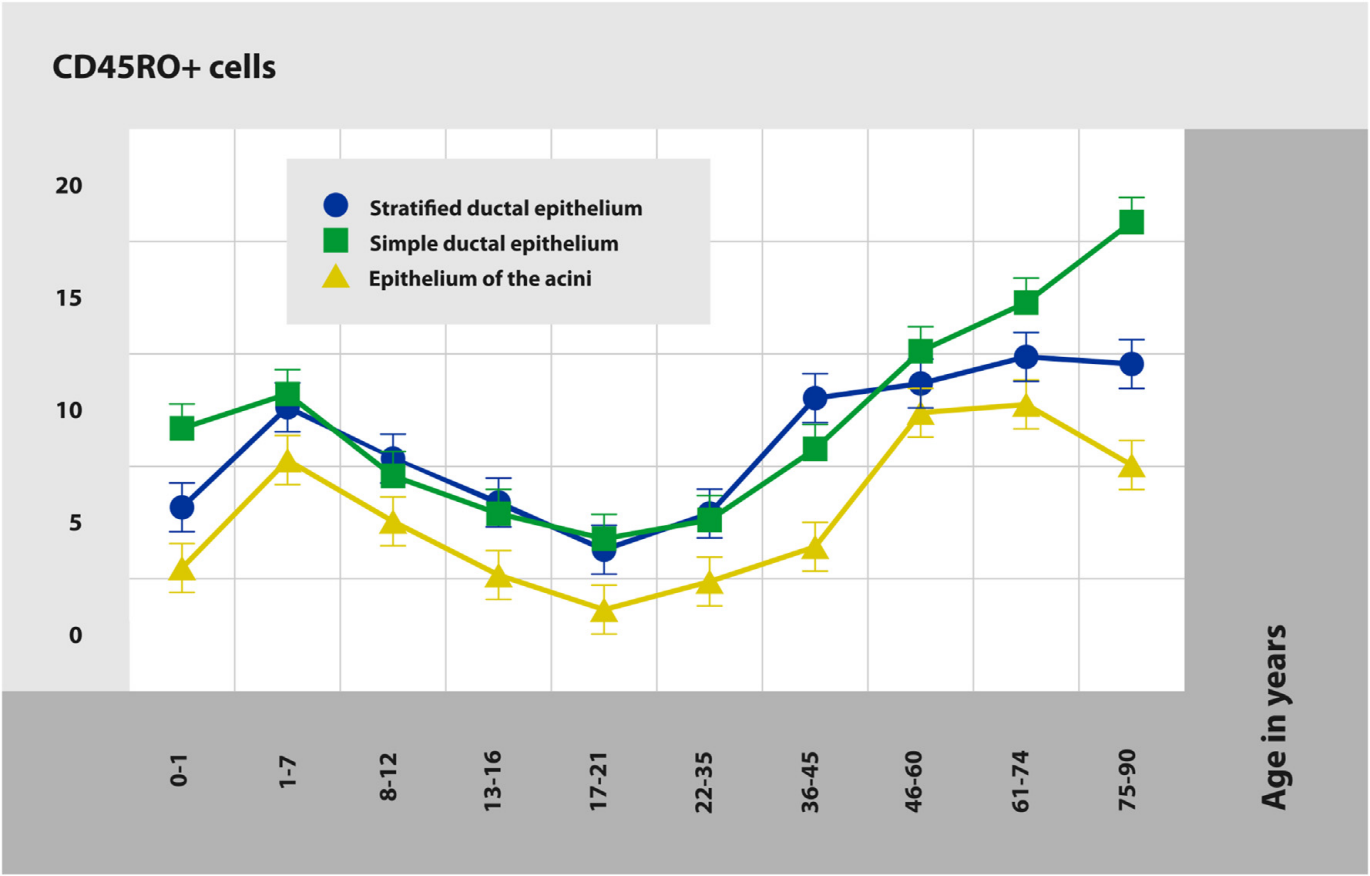
Distribution of CD45RO-positive cells in bulbourethral glands' epithelium in men of different age. The growth of CD45RO-positive cells amount is observed in the age periods of 1–7 years and from 36 to 90.

The relative amount of T cells in the bulbourethral glands varies with age (Figure 2). In ducts and acini, the number of CD45RO-positive cells increases in toddlers and young children (1–7 years old) comparing to the infancy age (10 days–1 year). Later, it gradually decreases both in late childhood (8–12 years) and in early adolescence (13–16 years), reaching the minimal values in late adolescents (17–21 years). In early adulthood (22–35 years), the number of T cells increases only in acini while in middle adults (a subgroup of 36–45 years), it also increases in ducts of the bulbourethral glands. In middle adulthood (a subgroup of 46–60 years) as well as in late adulthood (61–74 years) and in middle & oldest-old individuals (75–90 years), the number of CD45RO-positive cells in stratified epithelium of ducts remains without considerable changes, though in simple epithelium of ducts, it continues to grow. In middle adulthood, the number of T cells in secretory portions increases, but in late adults it does not change with a slight decrease in the oldest individuals.

**Figure 3:**
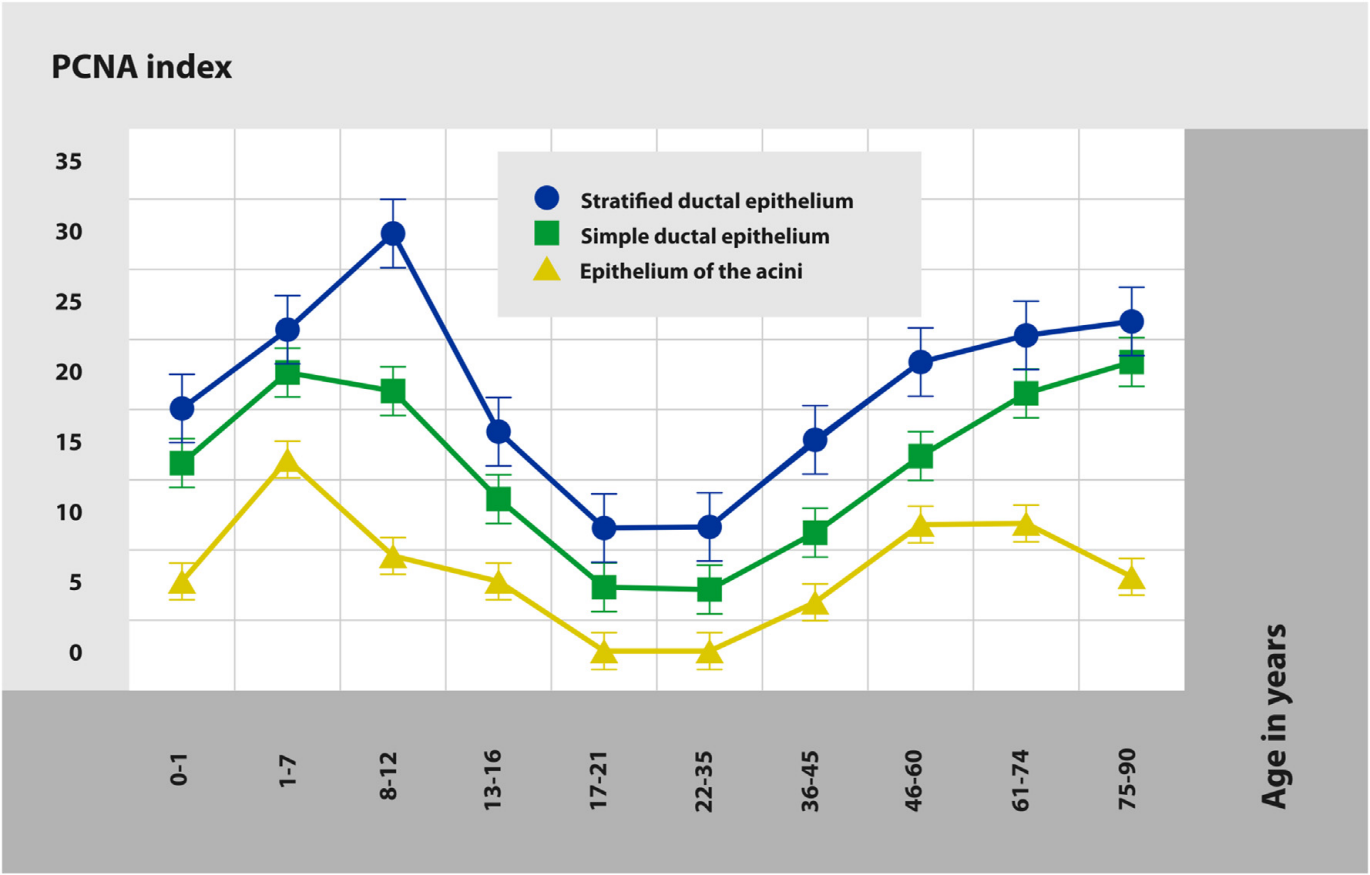
Distribution of PCNA-positive cells in bulbourethral glands' epithelium in men of different age. The growth of PCNA-positive cells amount is observed in the age periods of 1–12 years and from 46 to 90.

We have revealed the age-related dynamics of proliferative activity in the bulbourethral gland epithelium (Figure 3). Comparing to the infancy stage, PCNA indices in the ducts epithelium and in the secretory portions significantly increase in children aged from 1 to 7 years. In prepubertal stage (8–12 years), the proliferative activity of duct cells remains high, but decreases in acini. In early adolescents (13–16 years old), the index decreases markedly in all sections of the bulbourethral glands. In late adolescence (17–21 years) and in early adulthood (22–35 years), the proliferative activity of ductal epithelium is minimal being completely absent in acini. In middle adulthood (36–60 years), PCNA indexes of ductal epithelium and secretory portions increase again. In late adults (61–74 years) and the oldest men (75–90 years), the proliferative activity of duct epithelial cells still tends to increase while in secretory portions it remains the same in late adults with a slight decrease in the oldest group.

A comparison of the age-related changes in the number of T cells and proliferation indices has revealed a high degree of correlation of these parameters’ dynamics in all sections of the glands, i.e. in stratified epithelium of interlobular ducts and sinuses (r = 0.61), in simple epithelium of intralobular ducts (r = 0.76), and in secretory portions (r = 0.76).

## 4 Discussion

Data about T cell infiltration of the bulbourethral gland epithelium in adult men based on immunohistochemical studies are reported by some sources [13]. The results indicate that a presence of T cells in glandular epithelium is a feature of the bulbourethral gland morphofunctional status during the entire postnatal life. Moreover, in men of all the age groups a T cells predominance in ducts epithelium is found comparing to acini of the bulbourethral glands.

According to the mentioned study of lymphoid elements in the bulbourethral glands [13], intraepithelial lymphocytes reach 10% of the glandular cell population in men of 22–38 years old. Our quantitative analysis has revealed similar results for early adulthood (22–35 years); the total content of CD45RO-positive cells in duct epithelium and in acini of the bulbourethral glands is about 13%. At the same time, we demonstrate that the number of lymphoid cells in the gland parenchyma changes with age; it increases in children, gradually decreases by the youth period, and rises again since the age of middle adulthood.

Lymphoid elements of the bulbourethral glands are known to form a local immunological barrier, which is, however, less developed if compared to other reproductive organs [13]. Thus, immunocompetent cells number and diversity is much greater in the prostate. There are T and B cells, macrophages, and natural killers cells; most of them are activated [15, 17, 18, 19]. The authors consider the immunological barrier in the bulbourethral glands to be relatively weak due to low probability of tissues contact with sperm antigens there [13]. Our results show that the intensity of lymphocytic infiltration in the bulbourethral glands is changing, probably representing the intensity of barrier function at different periods of life. An increase in the number of intraepithelial lymphocytes in childhood may be regarded as an immunological barrier formation due to the onset of an urogenital tract colonization with diverse bacterial flora [20]. A persistent trend to gain the lymphoid elements of the bulbourethral glands in men after 36 years may be explained either by a rise of sperm antigenicity or by an increase in the amount of pathological spermatozoa, what is especially expressed in later periods of life [21, 22].

No studies on the proliferative activity of the human bulbourethral gland epithelium have been found in the available literature. Our analysis of PCNA-positive nuclei localization in the bulbourethral gland parenchyma convincingly shows that in all the studied age groups duct epithelial cells have higher proliferative activity than those of secretory portions. These data are consistent with the data about stem elements absence in acini of bulbourethral glands [23] suggesting their localization in ductal epithelium. Perhaps, poorly differentiated cells from the cambial zones of ducts are moving towards the acini causing the detection of PCNA-positive nuclei in glandular cells of newly formed acini located near the ducts or associated with them.

The revealed age-related dynamics of proliferative activity in the bulbourethral gland epithelium allows to assume that stem and poorly differentiated bulbourethral gland epithelial cells, as it goes in the prostate [24], are androgen-independent and are influenced by other regulatory factors, one of which might be represented by intraepithelial lymphocytes. A comparative analysis of the obtained data indicates that age-related changes in the PCNA indices of glandular cells, which demonstrate a negative correlation with the androgenization level of male body, positively correlate at the same time with fluctuations in the number of T cells in epithelium of ducts and of acini. In infancy, against the background of infantile release of testosterone [25], the values of PCNA indices and the number of T cells in the bulbourethral gland parenchyma are lower comparing to the subsequent period of childhood. An increase in proliferation of the bulbourethral gland epithelium in children (1–7 years), in the absence of endocrine activity of the testes, is accompanied by an increase in lymphocytic infiltration of ductal and acinar epithelium. In puberty, the value of cell proliferation in the bulbourethral glands decreases, giving way to androgen-dependent processes of differentiation and secretion of epithelial cells [5, 26], as evidenced by a progressive decrease in PCNA indices. At the same time, a decrease in CD45RO-positive cells number in ducts and secretory units is observed. During the period with the highest level of androgens in the body, namely in late adolescents and young adults [27], the PCNA indices are minimal. It might be interpreted as an evidence of a balance between proliferation and cell death, which is provided by androgens [5, 6]. Meanwhile, the minimal number of T cells in the gland parenchyma is registered.

An age-related activation of epithelial cell proliferation in the bulbourethral glands occurs against the background of a progressive decrease in the degree of male body androgenization. A drop in the level of circulating androgens disinhibits the apoptosis of differentiated glandular cells in the bulbourethral glands and disrupts cell homeostasis, stimulating cell multiplication [5, 6]. Moreover, assuming that cambial cells of bulbourethral glands (so as prostatic stem cells do) depend on estrogens, a progressive relative estrogenization in elderly men contributes to stimulation of epithelium proliferation [28, 29]. The hormone-triggered homeostatic mechanism requires the interaction of cell proliferation regulators, including probably T cells, the number of which increases in the bulbourethral glands in middle and late adulthood as well as in the old age.

In men aged 46 to 90, cambial epithelial cells of ducts and secretory portions show a different degree of dependence on the T cell presence. In stratified duct epithelium when there is an increase in proliferation index, the number of T cells does not change. So the lymphocytes perhaps do not contribute significantly to the mitotic activity stimulation in the epithelium. We consider that some other cell proliferation regulators may participate there (estrogens, growth factors of the stroma, hormones of diffuse endocrinocytes). In contrast, in simple epithelium of intralobular ducts, an increase in PCNA index is accompanied by an increase in the number of CD45RO-positive cells. In acini of bulbourethral glands (men aged 46–74 years), the proliferation index and the number of T cells remain unchanged. After that, they synchronously decrease in the middle & oldest-old period (75–90 years). It indicates a higher dependence of epithelial reproductive activity in intralobular ducts and secretory portions on the presence of T cells, but does not deny a participation of other cell proliferation stimulators. The fact that CD45RO can be detected on other immune cells may be used in future studies for further investigation.

## 5 Conclusions

The results of our study show that age-related fluctuations in the relative amount of T cells infiltrating the parenchyma of the bulbourethral glands correlate with changes in the level of proliferative activity for epithelial cells of excretory ducts and acini. We consider that T cells are involved in the regulation of cell proliferation in the bulbourethral gland epithelium, being most active when androgen levels in blood are low.

## Declarations

### Author contribution statement

Tatiana Boronikhina, Alexander Yatskovskiy: Conceived and designed the experiments; Analyzed and interpreted the data; Wrote the paper.

Gennadii Alexandrovich Piavchenko: Performed the experiments; Analyzed and interpreted the data; Wrote the paper.

Ekaterina Kustavinova: Performed the experiments; Contributed reagents, materials, analysis tools or data.

Natalia Kartashkina: Performed the experiments; Wrote the paper.

Artem Venediktov, Svetlana Mukhamedova, Igor Meglinski: Analyzed and interpreted the data; Wrote the paper.

Sergey Kuznetsov: Contributed reagents, materials, analysis tools or data; Wrote the paper.

### Funding statement

Dr. Gennadii Piavchenko acknowledges the support of Ministry of Science and Higher Education of the Russian Federation [075-15-2020-926]. Prof. Igor Meglinski acknowledges the partial support of innovative programme [101004462] and the Leverhulme Trust and The Royal Society [APX111232 APEX Awards 2021] and the Academy of Finland [325097]. Dr. Gennadii Piavchenko and Prof. Igor Meglinski also acknowledge the support from the Academy of Finland [351068].

### Data availability statement

Data will be made available on request.

### Declaration of interest’s statement

The authors declare no conflict of interest.

### Additional information

No additional information is available for this paper.
